# *Panax notoginseng* saponins ameliorate cisplatin‐induced mitochondrial injury via the HIF‐1α/mitochondria/ROS pathway

**DOI:** 10.1002/2211-5463.12760

**Published:** 2019-12-05

**Authors:** Qingqing Li, Xueyan Liang, Yufang Yang, Xian Zeng, Xiaobin Zhong, Chun Huang

**Affiliations:** ^1^ Department of Pharmacy The First Affiliated Hospital of Guangxi Medical University Nanning China; ^2^ Regenerative Medicine Research Center of Guangxi Medical University Nanning China

**Keywords:** cisplatin, HIF‐1α, mitochondria, *Panax notoginseng* saponins, ROS

## Abstract

Cisplatin is a major antineoplastic drug that is used to treat solid tumors, but its use is restricted by its nephrotoxicity. Such cisplatin‐induced nephrotoxicity (CIN) is believed to occur primarily through mitochondrial damage and reactive oxygen species (ROS) generation. Our previous studies have indicated that *Panax notoginseng* saponins (PNSs) mitigate CIN by enhancing hypoxia‐inducible factor 1α (HIF‐1α)‐induced mitochondrial autophagy. In this study, the role of the HIF‐1α/mitochondria/ROS pathway in PNSs protection against CIN was investigated using a rat model. A CIN model was generated by giving rats intraperitoneal injections with cisplatin (a single dose) and then treating them with or without 2‐methoxyestradiol (HIF‐1α inhibitor) and PNSs. We then measured ROS levels, superoxide dismutase, glutathione, catalase malondialdehyde and nitric oxide (to evaluate oxidative stress) and ATP, mitochondrial membrane potential and mitochondrial permeability transition pore opening (to evaluate mitochondrial function) in kidneys at different time points. We observed that PNSs remarkably reduced the levels of ROS, malondialdehyde and nitric oxide, as well as the opening of mitochondrial permeability transition pore, which is increased by cisplatin and further increased by HIF‐1α inhibition. In addition, PNSs increased the levels of superoxide dismutase, catalase and glutathione, as well as ATP and mitochondrial membrane potential in renal tissues; these are all reduced by cisplatin and further reduced by HIF‐1α inhibition. In conclusion, we demonstrate here that PNSs protects against mitochondrial damage induced by cisplatin through HIF‐1α/mitochondria/ROS.

## Graphical abstract

*Panax notoginseng* saponins are active ingredients extracted from *P. notoginseng*, which is an herb used in traditional Chinese medicine. Here, we demonstrate that *P. notoginseng* saponin protects against mitochondrial damage induced by cisplatin and decreases reactive oxygen species production through hypoxia‐inducible factor 1α/mitochondria/reactive oxygen species.

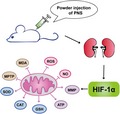

**Abbreviations**
2ME2: 2‐methoxyestradiol
CAT: catalase
CIN: cisplatin‐induced nephrotoxicity
GSH: glutathione
HIF‐1α: hypoxia‐inducible factor 1α
MDA: malondialdehyde
MMP: mitochondrial membrane potential
MPTP: mitochondrial permeability transition pore
NAG: *N*‐acetyl‐β‐d‐glucosaminidase
NO: nitric oxide
PNS: *Panax notoginseng* saponin
ROS: reactive oxygen species
SOD: superoxide dismutase
SD: standard deviation

Cisplatin is a major antineoplastic drug that is used to treat solid tumors. Despite its effectiveness, the application of cisplatin is restricted by its nephrotoxicity 1, 2. However, the molecular mechanism of cisplatin‐induced nephrotoxicity (CIN) has not yet been elucidated, and the effective therapeutic drug is still lacking in prevention and treatment of CIN. Recently, there have been growing pieces of evidence that mitochondrial dysfunction can increase the production of reactive oxygen species (ROS) 3, 4. Importantly, ROS can in turn damage mitochondria 5, which results in mitochondrial dysfunction 6. According to reports, cisplatin leads to releasing ROS and increasing oxidative stress 7, 8. Reducing the ROS production in renal tissue could protect kidneys from injury of oxidative stress in rats 9. Our previous research found that mitochondria are the most damaged organelles in CIN 10, 11. Therefore, mitochondrial damage and ROS‐mediated oxidative stress are thought to be the major mechanisms in CIN 12, 13.

*Panax notoginseng* saponins (PNSs), extracted from *Panax notoginseng*, have been used to treat cardiovascular and cerebrovascular diseases for many years. PNSs have a variety of pharmacological effects 14, such as inhibiting tumor growth 15, antioxidant effects 16 and antiproliferative effects, among others. Some evidence showed that PNSs can protect mice or cells from oxidative stress injury 17, 18 and restore mitochondrial membrane potential (MMP), which helps to reduce the damage caused by Alzheimer’s disease 19. Our previous studies showed that PNSs could protect against CIN 10, 11, 20. Therefore, PNSs can protect against CIN and do not weaken the antitumor effect of cisplatin 15, 21.

Our previous studies found that PNSs could protect from CIN by enhancing hypoxia‐inducible factor‐1α (HIF‐1α)‐mediated mitochondrial autophagy 11. However, the relationships among the mitochondria, ROS and HIF‐1α are unclear. In this study, we speculate that the HIF‐1α/mitochondria/ROS pathway plays an important role in PNSs protecting rats from CIN.

## Materials and methods

### Chemicals

Powder injection of cisplatin (batch no. 5050272DB) was purchased from Qilu Pharmaceutical Co., Ltd. (Jinan, Shandong, China). 2‐Methoxyestradiol (2ME2; batch no. 105M4158V) was obtained from Sigma‐Aldrich Co. (St. Louis, MO, USA). Powder injection of PNSs (batch no. 14092108) was obtained from Wuzhou Pharmaceutical Co., Ltd. (Wuzhou, Guangxi, China). Kits of superoxide dismutase (SOD; batch no. A001‐1), malondialdehyde (MDA; batch no. A003‐1), nitric oxide (NO; batch no. 20170605), glutathione (GSH) and catalase (CAT; batch no. 20170608) were from Nanjing Jiancheng Bioengineering Research Institute (Nanjing, Jiangsu, China). Kits of ROS (batch no. S0033) and ATP (batch no. S0026) were obtained from Beyotime Technology Co., Ltd. (Shanghai, China). MMP kit (batch no. 20170301) was purchased from Beijing Solarbio Science & Technology Co., Ltd. (Beijing, China). Mitochondrial permeability transition pore (MPTP) Fluorescence Assay Kit (batch no. GMS10095.2) was obtained from GENMED Scientifics, Inc. (Boston, MA, USA).

### Animals

Male Sprague–Dawley rats (200 ± 20 g) were obtained from the Experimental Animal Center of Guangxi Medical University (Guangxi, China). The rats were housed five per cage at 25 ± 5 °C and 60 ± 20% humidity, and with a standard diet and water *ad libitum*. The experiments were managed based on the protocols approved by the Animal Experimental Ethical Committee of Guangxi Medical University (approval no. 201310009).

### **Experimental design and drug administration** 11

After acclimatization for a week, the rats were divided randomly into five groups as in our previous study 11: (a) the control group (with the same volume of saline as PNSs from days 1 to 3 and the same volume of saline as cisplatin on day 1); (b) the cisplatin group [with a single dose of cisplatin (5 mg·kg^−1^) on day 1 and the same volume of saline as PNSs from days 1 to 3]; (c) the cisplatin + PNS group [with a single dose of cisplatin (5 mg·kg^−1^) on day 1 and PNSs (31.35 mg·kg^−1^) from days 1 to 3]; (d) the 2ME2 + cisplatin group [with a single dose of 2ME2 (4 mg·kg^−1^), 1.5 h later with a single dose of cisplatin (5 mg·kg^−1^) on day 1, and then the same volume of saline as PNSs from days 1 to 3]; and (e) the 2ME2 + cisplatin + PNS group [with a single dose of 2ME2 (4 mg·kg^−1^), 1.5 h later a single dose of cisplatin (5 mg·kg^−1^) on day 1, and then with PNSs (31.35 mg·kg^−1^) from days 1 to 3].

All drugs were administered by abdominal cavity injection. The doses of cisplatin and PNSs came from our previous study 10, 20. The 2ME2 dose came from previous reports 22, 23.

### **Specimen collection** 11

Urine, blood and kidney specimens were collected after rats were exposed to cisplatin for 3, 6, 12, 24 and 72 h. In brief, urine and blood specimens were collected for detecting the levels of urinary *N*‐acetyl‐β‐d‐glucosaminidase, blood urea nitrogen and serum creatinine. After urine and blood specimens were collected, the rats were sacrificed by injecting sodium pentobarbital intraperitoneally (30 mg·kg^−1^), and the renal specimen was washed with ice‐cold saline and removed. Part of the renal specimen was fixed for hematoxylin and eosin staining and transmission electron microscopy 11; the rest of the renal specimen was stored at −80 °C immediately for further analysis.

The results of serum creatinine, blood urea nitrogen and urinary *N*‐acetyl‐β‐d‐glucosaminidase levels, as well as the results from renal pathological examination using hematoxylin and eosin staining and transmission electron microscopy, indicated that cisplatin induced renal damages. Moreover, PNSs had a protective effect on CIN 11.

### The determination of ROS

Reactive oxygen species was determined by a fluorescent probe 2′, 7′‐dichlorodihydrofluorescein diacetate using the ROS assay kit (Beyotime, China) based on the manufacturer’s directions. In brief, the renal tissues of rats were homogenized with 0.9% NaCl solution and centrifuged at 500 ***g*** for 10 min at 4 °C. After removing the supernatant, the pelleted materials were suspended in saline; then 2′,7′‐dichlorodihydrofluorescein diacetate (1 : 1000) was added. Next, the contents were mixed and incubated at 37 °C in the dark for 30 min. Finally, the fluorescence intensity was measured on a Multi‐Mode Microplate Reader (Synergy H1, Winooki, VT, USA) with excitation and emission wavelengths of 485 and 528 nm, respectively. The results were presented as the fluorescent intensity per nanogram of protein.

### MDA and NO determination

In brief, 10% renal tissue homogenate was prepared with 0.9% NaCl solution using a homogenizer. A part of the homogenate was centrifuged at 1409 ***g***. for 20 min at 4 °C, and the supernatant was used to measure MDA level by UV‐Vis spectrophotometer (TU‐1901; PERSEE, Beijing, China) at 532 nm. The remaining homogenate was centrifuged at 978 ***g***. for 10 min at 4 °C; then the NO level in the supernatant was determined using Microplate Reader (Spectra Max Plus 384; Molecular Devices Limited, Hongkong, China) at 550 nm.

### Superoxide dismutase, GSH and CAT assay

In brief, after 10% homogenate of kidney was prepared in 0.9% NaCl solution, a part of the homogenate was centrifuged for 20 min at 1409 ***g***. and at 4 °C; then the activity of SOD in the supernatant was measured. The remaining homogenate was centrifuged at 978 ***g***. for 10 min at 4 °C; then the activity of CAT and the concentration of GSH in the supernatant were determined.

### The extraction of mitochondria

Purified mitochondria were obtained using Mitochondrial Extraction Kit (Solarbio) based on the manufacturer’s instructions. In brief, the renal tissues of rats were homogenized by lysis buffer and centrifuged for 5 min at 1000 ***g*** at 4 °C. After the supernatant was centrifuged for 10 min at 12 000 ***g***, the pelleted materials were washed with wash buffer and resuspended in store buffer. The protein concentration was measured by BCA Protein Assay Kit (Beyotime, Nanjing, China) based on the manufacturer’s protocol. The isolated mitochondrial fraction was collected for the assays of MMP, ATP level and MPTP.

### Determination of ATP level in mitochondria

ATP level was detected by the ATP assay kit (Beyotime) based on the manufacturer’s instructions. In brief, after dissolved by lysis buffer, the isolated mitochondria were centrifuged at 12 000 ***g*** for 10 min at 4 °C. Then, the supernatant was mixed with ATP reagents. ATP level was measured by a Multi‐Mode Microplate Reader (Synergy H1). Finally, an ATP standard curve was established; then the ATP level was calculated.

### MMP determination

MMP was measured by a fluorescent probe JC‐1 using the MMP assay kit (Solarbio) based on the manufacturer’s directions. In brief, the isolated mitochondria were added into JC‐1 staining working solution. After the contents were mixed, the fluorescence intensity of both mitochondrial JC‐1 monomers (green fluorescence; excitation wavelength (λex) 490 nm, emission wavelength (λem) 530 nm) and aggregates (red fluorescence; λex 525 nm, λem 590 nm) were measured by a Multi‐Mode Microplate Reader (Synergy H1). The MMP was calculated according to the fluorescence ratio of red to green per milligram of protein.

### Determination of MPTP opening

The MPTP opening was detected by MPTP Fluorescence Assay Kit (Genmed) according to the manufacturer’s instructions. After centrifugation for 5 min at 16 000 ***g*** at 4 °C, the isolated mitochondrial suspension was mixed with the staining working solution, which contained staining solution (reagent A) and neutralization solution (reagent B). Next, the contents were incubated at 37 °C in the dark for 15 min. At last, the fluorescence intensity was detected on a Multi‐Mode Microplate Reader (Synergy H1) with excitation and emission wavelengths of 488 and 505 nm, respectively. Results were presented as relative fluorescence intensity (fluorescence intensity per milligram of protein).

### Statistical analysis

The quantitative data were shown as the mean ± standard deviation (SD). Statistical analysis was performed using the spss 19.0 software for Windows (SPSS Inc., Chicago, IL, USA). The difference between groups was analyzed by one‐way ANOVA. A *P*‐value  <0.05 was considered to be statistically significant.

## Results

### PNSs reduced the levels of ROS, MDA and NO in kidney tissues induced by cisplatin and 2ME2

**Figure 1:**
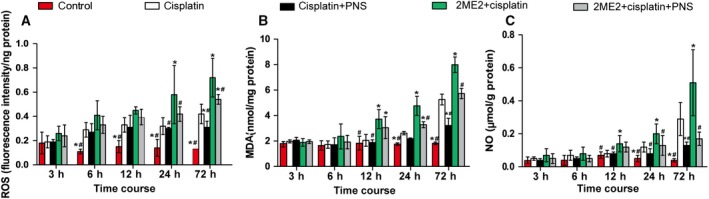
The levels of ROS (A), MDA (B) and NO (C) in renal tissues from rats at each time point. *P < 0.05 compared with the cisplatin group; ^#^ *P* < 0.05 compared with the 2ME2 + cisplatin group. Data were indicated as mean ± SD (*n* = 6) and analyzed by one‐way ANOVA.

The rats in the cisplatin group demonstrated higher ROS (Fig. 1A) (from 6 to 72 h), MDA (Fig. 1B) and NO (Fig. 1C) (24 and 72 h) levels when compared with the control group (Fig. 1). The 2ME2 + cisplatin group rats showed further increased ROS (24 and 72 h), MDA and NO (12, 24 and 72 h) levels compared with the cisplatin group (Fig. 1). However, the rats in the cisplatin + PNS group showed lower ROS (73.81%), MDA (61.22%) and NO (44.83%) levels compared with the cisplatin group in 72 h, and the 2ME2 + cisplatin + PNS group rats showed lower ROS levels (75% and 72.41% in 24 and 72 h), MDA (71.71% in 72 h) and NO (33.33% in 72 h) compared with the 2ME2 + cisplatin group (Fig. 1).

In addition, with the extension of time, the changes of ROS, MDA and NO levels in rat renal tissues from each group were shown in Fig. 1.

### PNSs increased the levels of SOD, CAT and GSH in kidney tissues reduced by cisplatin and 2ME2

**Figure 2:**
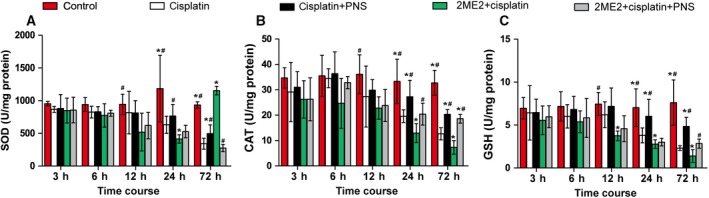
The levels of SOD (A), CAT (B) and GSH (C) in renal tissues from rats at each time point. *P < 0.05 compared with the cisplatin group; ^#^ *P* < 0.05 compared with the 2ME2 + cisplatin group. Data were indicated as mean ± SD (*n* = 6) and analyzed by one‐way ANOVA.

The rats in the cisplatin group demonstrated lower SOD (Fig. 2A), CAT (Fig. 2B) and GSH (Fig. 2C) levels (24 and 72 h) compared with the control group (Fig. 2). The 2ME2 + cisplatin group rats showed further decreased SOD, CAT (24 and 72 h) and GSH levels (12, 24 and 72 h) compared with the cisplatin group (Fig. 2). However, the cisplatin + PNS group rats showed higher SOD (1.45‐fold in 72 h), CAT (1.39‐ and 1.61‐fold in 24 and 72 h) and GSH (1.58‐ and 2.09‐fold in 24 and 72 h) levels compared with the cisplatin group, and the 2ME2 + cisplatin + PNS group showed higher SOD (1.79‐fold in 72 h), CAT (1.57‐ and 2.61‐fold in 24 and 72 h) and GSH (2.04‐fold in 72 h) levels compared with the 2ME2 + cisplatin group (Fig. 2).

In addition, with the extension of time, the changes in SOD, CAT and GSH levels in rat renal tissues from each group were shown in Fig. 2.

### PNSs increased the levels of ATP and MMP in kidney tissues reduced by cisplatin and 2ME2

**Figure 3:**
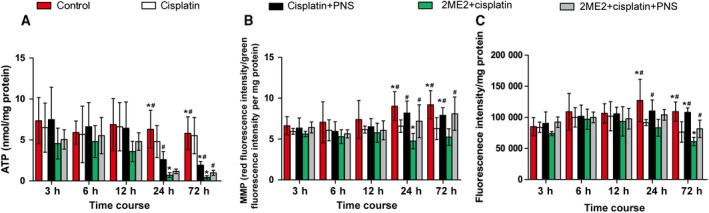
The levels of mitochondrial ATP (A) and MMP (B) and the relative fluorescence intensity of MPTP opening (C) in renal tissues from rats at each time point. *P < 0.05 compared with the cisplatin group; ^#^ *P* < 0.05 compared with the 2ME2 + cisplatin group. Data were indicated as mean ± SD (*n* = 6) and analyzed by one‐way ANOVA.

The rats in the cisplatin group demonstrated lower ATP (24 and 72 h) and MMP levels compared with the control group (Fig. 3A,B). The 2ME2 + cisplatin group rats showed further decreased ATP (24 and 72 h) and MMP levels (Fig. 3A,B) compared with the cisplatin group. However, the cisplatin + PNS group rats showed higher ATP (2.14‐fold) and MMP (1.28‐fold) levels compared with the cisplatin group in 72 h, and the 2ME2 + cisplatin + PNS group rats showed higher ATP (2.2‐fold) and MMP (1.44‐fold) levels in 72 h compared with the 2ME2 + cisplatin group (Fig. 3A,B).

In addition, with the extension of time, the changes of ATP and MMP levels in rat renal tissues from each group were shown in Fig. 3A,B.

### PNSs decreased MPTP in kidney tissues induced by cisplatin and 2ME2

The expression of MPTP was displayed using MPTP fluorescence assay when the stronger the fluorescence intensity, the lower the MPTP opening (see details in Fig. 3C). The rats in the cisplatin group demonstrated higher MPTP levels (24 and 72 h) compared with the control group. The 2ME2 + cisplatin group rats showed further increased MPTP levels (72 h) compared with the cisplatin group. However, the cisplatin + PNS group rats showed lower MPTP levels (71.43% in 72 h) compared with the cisplatin group, and the 2ME2 + cisplatin + PNS group also showed lower MPTP levels (74.62% in 72 h) compared with the 2ME2 + cisplatin group.

In addition, with the extension of time, the change of MPTP level in rat renal tissues from each group was shown in Fig. 3C.

## Discussion

Cisplatin is a cell‐cycle nonspecific drug, so cisplatin has more side effects. Cisplatin‐induced nephrotoxicity (CIN) is one of the main side effects that limit its clinical application. According to reports, cisplatin caused kidney injury through the induction of ROS and oxidative stress 24, 25. Reduced ROS resulted in antiapoptosis and antioxidative stress 25. In addition, damaged mitochondria can produce ROS in large quantities. Mitochondrial damage and ROS generation are thought to be the major factors promoting the occurrence and development of CIN 12, 13, 26. Moreover, our previous research showed that cisplatin could induce renal damage 10, 11, 20, which was aggravated by specific inhibition of HIF‐1α (2ME2) 11. Importantly, PNS has a protective effect on CIN 10, 11, 20 by enhancing HIF‐1α‐mediated mitochondrial autophagy. However, the relationships among mitochondria, ROS and HIF‐1α are unclear. In this study, we examined the role of the HIF‐1α/mitochondria/ROS pathway in PNS protection from CIN.

It has been found previously that cisplatin increased the levels of ROS 27, MDA and NO in kidneys, whereas it decreased antioxidant enzyme activities of SOD, CAT and GSH 28. Notably, the overproduction of ROS induced by cisplatin increases the MDA level (a lipid peroxidation marker) in kidneys 29, 30, which probably resulted in the consumption of endogenous antioxidants such as GSH, SOD and CAT 29, 31. These imbalances of oxidative and antioxidative status resulted in oxidative stress injury of kidney 32, 33. PNSs is the active ingredient extracted from *Panax notoginseng*, which is a traditional Chinese medicine herb. Evidence has shown that PNSs decreased mitochondrial ROS, MDA and NO levels, whereas it increased the activities of SOD and GSH to protect from oxidative stress in retinal capillary endothelial cells 17. In our study, PNSs decreased the ROS and MDA levels, which were increased by cisplatin and were further increased by inhibition of HIF‐1α. In addition, PNSs significantly increased the levels of SOD, CAT and GSH, which were decreased by cisplatin and were further reduced by inhibition of HIF‐1α. These results indicate that PNS reduces cisplatin‐induced oxidative stress through the HIF‐1α pathway.

Cisplatin can accumulate in mitochondria and cause mitochondrial dysfunction 34. It was reported that cisplatin increased the opening of MPTP 35 and decreased the levels of ATP and MMP, which resulted in mitochondrial dysfunction in epithelial cells of renal tubules 36. In addition, PNSs could protect the mitochondrial function of rat cardiac myocytes by enhancing MMP 37. A recent report showed that PNSs protected kidney from diabetes by activating antioxidant proteins 38. In this study, PNSs significantly increased ATP and MMP levels, which were decreased by cisplatin and were further decreased by inhibition of HIF‐1α. In addition, PNSs significantly reduced the MPTP opening, which was increased by cisplatin and further increased by HIF‐1α inhibition. Our results suggest that PNSs protects mitochondrial dysfunction induced by cisplatin through the HIF‐1α pathway.

### Relationship between ROS and mitochondria

As is commonly known, ROS are mainly produced in mitochondria 3, 4, 39. Mitochondrial dysfunction can increase ROS generation 3, 4. Importantly, ROS overexpression can in turn damage mitochondrial DNA and the oxidative respiratory chain 5, which ultimately aggravates mitochondrial dysfunction. Mitochondrial membrane injury was a result of ROS production and lipid peroxidation, resulting in MPTP opening and reduction of MMP 40. Therefore, mitochondria are the main targets of oxidative stress, and ROS are the triggers of mitochondrial dysfunction 6. Based on the earlier described results of this study, we know that cisplatin‐induced mitochondrial damage significantly increased ROS level and activated oxidative stress. PNSs could protect the mitochondria by reducing ROS and oxidative stress.

### Relationship between ROS and HIF‐1α‐mediated mitophagy

**Figure 4:**
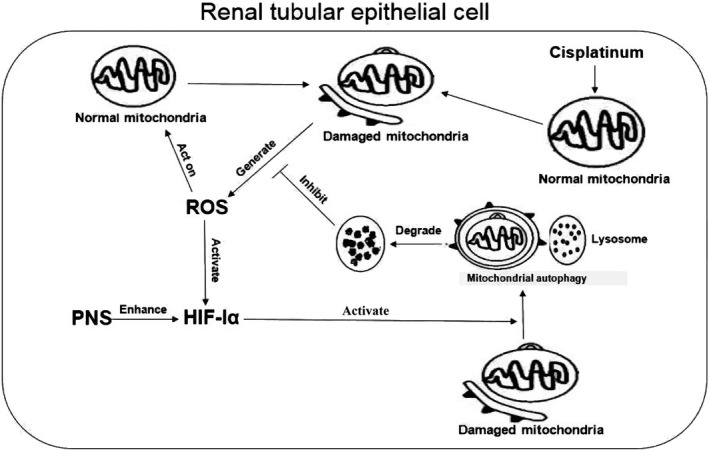
Cisplatin induced mitochondria injury and released ROS, which in turn damaged the mitochondria. PNSs enhanced HIF‐1α‐mediated mitochondrial autophagy to selectively remove damaged mitochondria, which resulted in reduced ROS production.

Mitophagy is a special form of autophagy that can selectively remove unwanted and damaged mitochondria 41, 42. Mitochondrial quality is a crucial determinant of cell fate, in which mitophagy plays a central role 43 following stresses such as ROS production 42. Therefore, mitophagy can reduce cell damage to promote cell survival by decreasing the high ROS level 44. In addition, elevated mitochondrial ROS activated HIF‐1α level and increased its target genes BNIP3 45, which induced mitophagy 46. Moreover, our previous study showed that PNSs protected CIN through enhancing HIF‐1α‐mediated mitophagy 11. The expression of HIF‐1α was increased on mRNA level after the treatment of PNSs. In this study, we found that PNSs improved the mitochondrial dysfunction and decreased ROS level that were induced by cisplatin and aggravated by inhibition of HIF‐1α. Combined with the results of our previous studies 11, we demonstrated that the damaged mitochondria, induced by cisplatin, produce and release ROS in large quantities. The ROS accumulation activated HIF‐1α and subsequently triggered mitophagy 11, which removed the damaged mitochondria. PNSs can enhance the HIF‐1α‐mediated mitophagy and allow the renal tubular epithelial cells to survive (Fig. 4).

In summary, we first demonstrated that PNSs could attenuate mitochondrial dysfunction and decrease ROS production by the HIF‐1α/mitochondria/ROS pathway.

## Conflict of interest

The authors declare no conflict of interest.

## Author contributions

YY and XZ conceived and designed the project. QL, XL and XZ acquired the data. QL and CH analyzed and interpreted the data. QL and YY wrote the paper.
